# Designing a Pilot Study Protocol to Test a Male Alcohol Use and Intimate Partner Violence Reduction Intervention in India: Beautiful Home

**DOI:** 10.3389/fpubh.2018.00218

**Published:** 2018-08-07

**Authors:** Miriam A. Hartmann, Saugato Datta, Rachel F. Banay, Vivien Caetano, Rosii Floreak, Prarthana Appaiah, Anuradha Sreevasthsa, Susan Thomas, Sumithra Selvam, Quinn Barnette, Krishnamachari Srinivasan

**Affiliations:** ^1^Women's Global Health Imperative, RTI International, San Francisco, CA, United States; ^2^Ideas42, New York, NY, United States; ^3^Division of Mental Health & Neurosciences, St. John's Research Institute, Bengaluru, India

**Keywords:** intimate partner violence, alcohol, breathalyzer, contingency management, behavioral economics, cognitive behavioral therapy, behavioral couple therapy

## Abstract

**Introduction:** Evidence suggests alcohol consumption is correlated with intimate partner violence (IPV) making alcohol reduction interventions a promising method for reducing IPV. While both financial incentive and cognitive behavioral therapy (CBT) interventions in high-income countries, respectively, have effectively reduced alcohol consumption and IPV perpetration among men, little evidence exists demonstrating that these approaches can work in a low-resource setting.

**Methods:** The objective of this study is to design and pilot test a low-cost, scalable intervention for reducing alcohol consumption and IPV in Bengaluru, India, where alcohol has been shown to be a key driver of high rates of IPV. A pilot randomized controlled trial (RCT) design will be used to examine the feasibility of testing a combined incentive and CBT based intervention among couples to stimulate immediate behavior change and to sustain positive behaviors pertaining to alcohol use and IPV. Sixty couples will be screened and enrolled into one of three study arms: an incentive-only, incentive plus counseling, or a control arm. Extensive procedures have been included to ensure participant safety, including staff training on global safety procedures for violence intervention research, careful messaging of study aims, screening procedures to exclude those at high risk of alcohol withdrawal or severe violence due to the study, and a referral and case management system. Male and female participants will complete surveys at baseline and immediately and 3-months post-intervention. Breathalyzers will be used to capture male participants' blood alcohol content daily for intervention arm participants and three times a week for control participants. A sub-sample of male and female members of couples will participate in qualitative in-depth interviews to further explore pathways to change. The results from this preliminary study will inform the development of a larger RCT study of male alcohol and IPV reduction.

## Introduction

Both experimental and correlational evidence indicate that alcohol consumption can increase the risk of intimate partner violence (IPV). A meta-analytic review of the evidence demonstrates that the association between alcohol consumption and IPV remains significant even when controlling for potential confounding variables, suggesting that at least a portion of IPV risk can be directly attributed to alcohol misuse (1). This link persists when controlling for hostility, antisocial behavior, and norms surrounding aggression (2–4).

Several hypotheses may explain the association between alcohol consumption and IPV; some studies argue that the lowered inhibitions and distorted perceptions of cues resulting from alcohol intoxication may lead to increased aggression (5–7). The Alcohol Myopia Model (AMM), on the other hand, posits that alcohol consumption has a myopic effect on attention, leading intoxicated individuals to focus on the most salient (often provocative) cues in a hostile situation, rather than subtler inhibitory cues (6).

As a result, alcohol reduction interventions are a promising channel for combatting IPV. Several evidence-based individual and couple-level interventions offer potential cost-effective solutions. In particular, there is a large body of evidence supporting the effectiveness of small financial incentives in promoting behavior change in low-resource settings, including India, Mexico, and Tanzania (8–10). In this approach, called contingency management, financial incentives reduce alcohol use by giving drinkers a short-term financial benefit in exchange for abstaining from alcohol use. These financial incentives aim to offset the immediate benefits drinkers might experience from consuming alcohol and make the long-term benefits of avoiding harmful drinking behavior (or conversely the negative consequences of engaging in harmful drinking) salient in the present. Schilbach (10) found that small incentives given to rickshaw drivers in Chennai, India both significantly reduced daytime drinking and increased savings by 60%. In Schilbach's experiment, 229 rickshaw drivers were randomized to one of several conditions involving financial incentives over the course of 3 weeks. Sobriety was measured using a daily breathalyzer test taken at the study office. The study found reduced daytime drinking (i.e., around the time of the breathalyzer tests), however, because drinking at other times increased, overall no reduction in alcohol consumption was observed.

Although small incentives can create short-term behavior change, they do not seem to create long-term change (11). However, when combined with behavioral couples' therapy (BCT) [i.e., couples counseling that uses cognitive behavioral therapy (CBT) as its base] designed to increase sobriety, provide men with alcohol reduction tactics and improve couples' communication (12), an intervention may have more sustained results for reducing IPV and alcohol use. A study in Liberia testing a CBT intervention compared to a CBT and cash-incentive intervention among criminally engaged men found effects were sustained longer among those receiving the combined intervention of cash incentive and CBT (13).

While couples counseling for IPV reduction has historically been controversial, in part due to concerns around safety, a recent call has been made to re-examine this approach, particularly in low-income settings where women have fewer options to leave (14, 15). A systematic review of couples-based therapy for IPV reduction concluded that this approach can be effective, particularly in cases of situational violence and where couples are committed to staying together (16). Of note, several studies included in the review compared BCT with other counseling and intervention approaches among couples where one was using substances. They found that couples receiving BCT reported lower levels of violence at follow-up than participants in other intervention arms (17, 18).

Though the bulk of research on CBT for alcohol and violence reduction has been conducted in high-income countries (19), in part due to a lack of high-skilled counselors in low-resource settings and the cost of training such counselors, one study in India suggests that this counseling can be feasibly conducted by trained lay counselors (20). A second study in India suggests this approach may be effective in reducing IPV perpetration among men with harmful drinking (21). In this latter study, of 177 alcohol dependent men who screened positive for IPV perpetration in Bengaluru, those who were randomized to receive CBT had significantly lower levels of IPV perpetration 3 months following the intervention.

Together, these bodies of work suggest a promising avenue for reducing IPV in resource-constrained settings. Thus, in this pilot study using a randomized controlled design we intend to examine the feasibility of testing a combined incentive and BCT based intervention to stimulate immediate behavior change and to sustain positive behaviors pertaining to alcohol use and IPV, which will inform the development of a larger RCT study (22).

## Materials and methods

### Objectives

Given the lack of existing rigorous research on reducing alcohol use and IPV in developing countries, we aim to begin filling the gap by conducting our work in an urban slum settlement in Bengaluru, India. Feasibility of the study will be evaluated through the following specific objectives:

1. Ability to recruit, enroll and retain couples in the two intervention arms and the control arm;
2. Ability of male participants to consistently use breathalyzers as a measure of sobriety; and
3. Couple's level of engagement with BCT sessions.

We will also assess preliminary effects of the intervention on primary outcomes including household safety and male partner sobriety, as well as on intermediate outcomes including attitudes and behaviors related to gender equality and shared household decision-making, which are relevant to longer-term outcomes of IPV prevention.

### Study design

**Figure 1:**
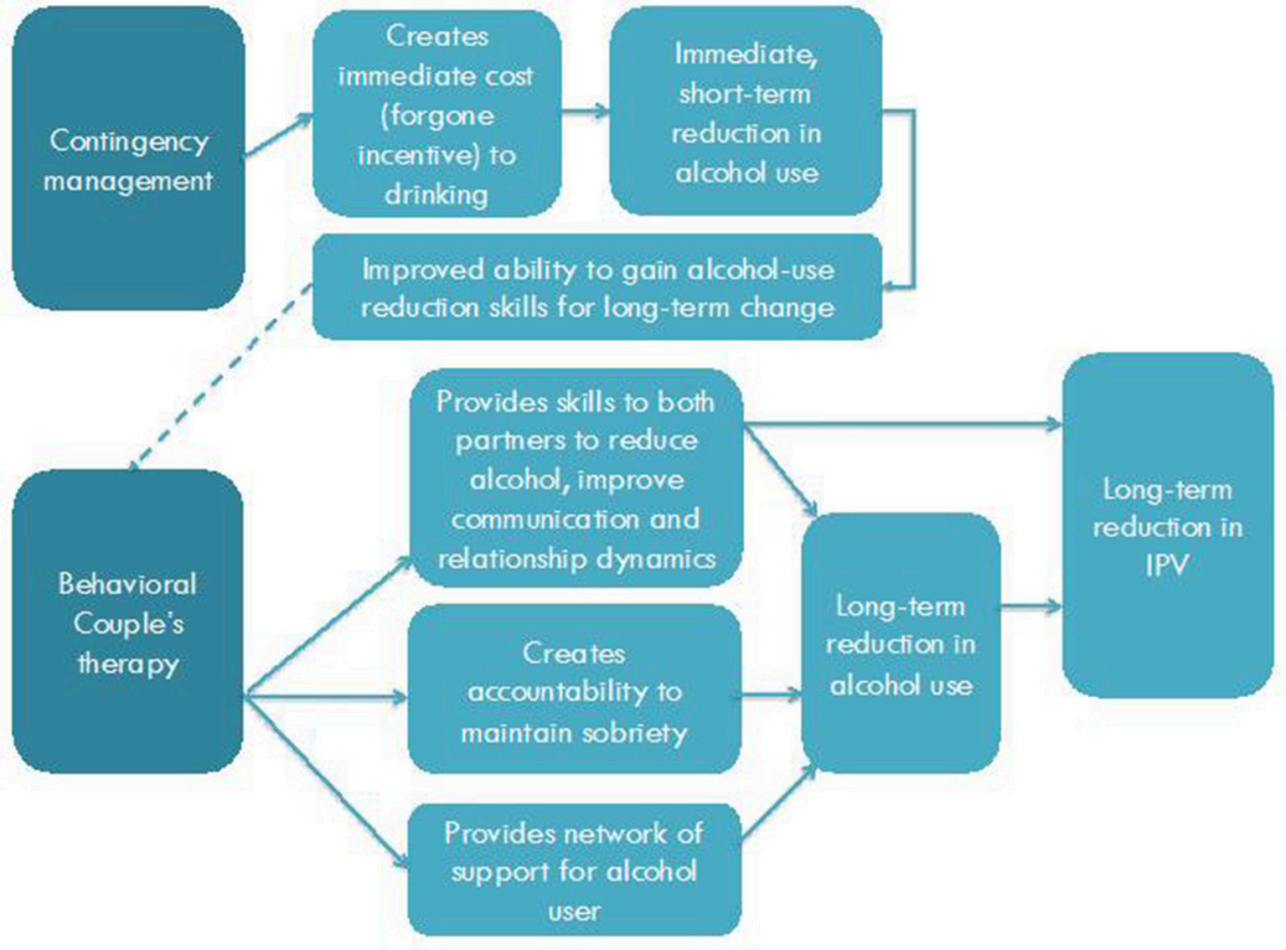
*Beautiful Home* intervention Theory of Change (TOC).

This study is a randomized control pilot study of *Beautiful Home*, an innovative combined incentive-based and BCT intervention developed with input from community partners for the mitigation of male alcohol use and IPV among couples in a slum in Bengaluru, India. Figure 1 depicts our Theory of Change (TOC). We hypothesize that the *Beautiful Home* intervention is feasible and can be delivered safely, leading to improvements in short-term alcohol reduction as a result of financial incentives. When combined with BCT, we posit the intervention will enable couples to learn skills that will support longer-term change related to alcohol use and IPV. Based on the results of this pilot, we will refine our approach, and determine the merit of a future phase 3 trial of the intervention.

**Figure 2:**
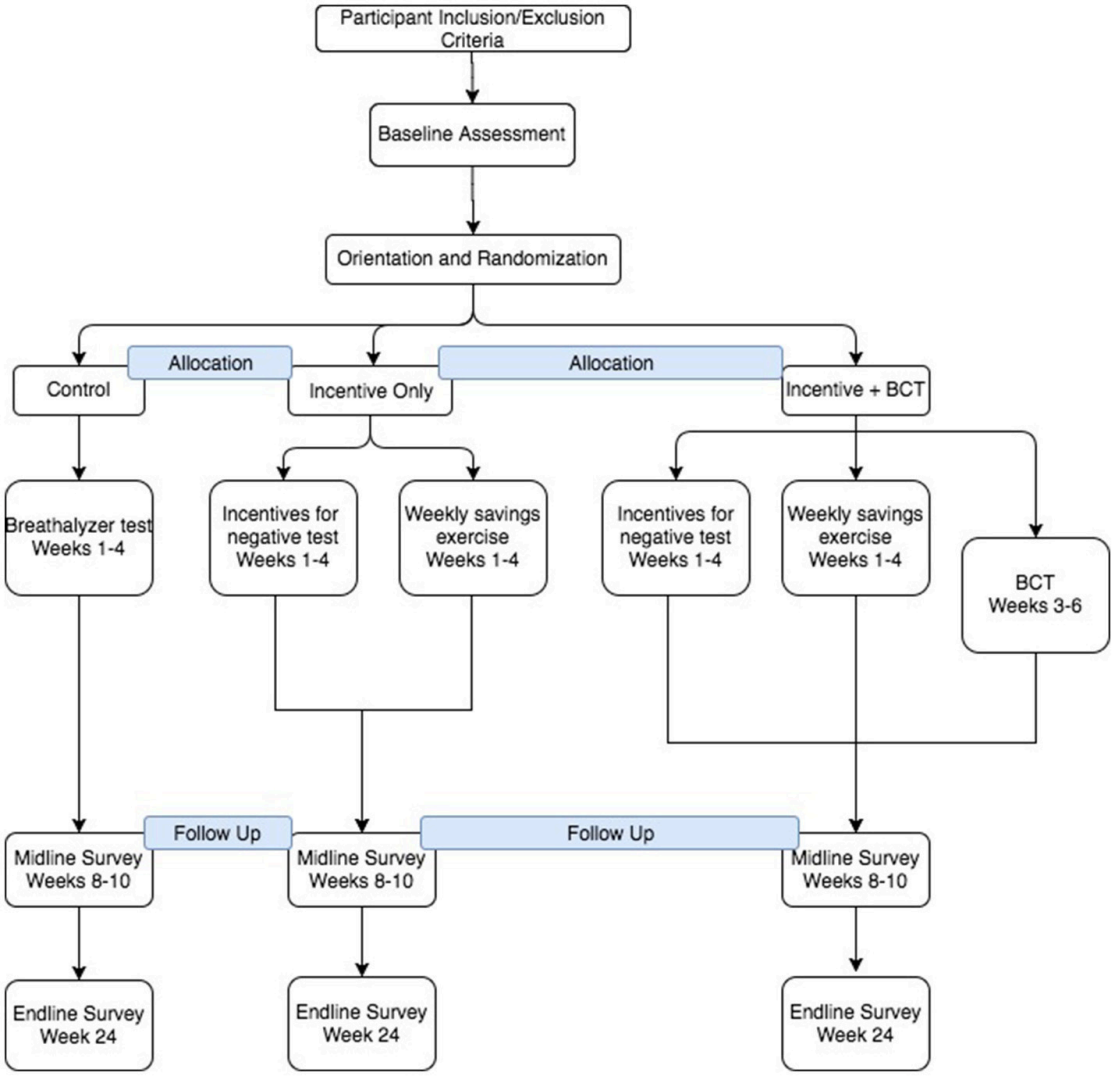
Flow diagram of the progress through the phases of the 3-arm randomized trial of enrollment, allocation to intervention arm, and data collection points.

The study will be conducted with 60 couples randomized into one of three arms: an incentive-only arm, an incentive-plus-counseling arm, and a comparison arm. The design mimics the proposed design of a follow-on randomized control trial, and allows for testing the feasibility and acceptability of the study design, as well as offering an opportunity to determine whether the combined approach of incentives and counseling performs better than an incentive-only intervention. During the intervention, breathalyzer data will be collected daily for the first 4 weeks from men using Soberlink© breathalyzer technology that allows for photo capture to ensure the appropriate person is taking the test. A period of 4 weeks was chosen for the incentives portion of the intervention based on the duration of Schilbach's study (10) and to preserve the team's flexibility to adapt if the initial incentive structure was not affecting drinking. Participants in the incentive-plus-counseling arm will participate for an additional 2 weeks (6 weeks) to accommodate counseling sessions. No breathalyzer data will be collected for the additional 2 weeks. Women and men will complete a baseline survey, as well as surveys immediately and 3 months post-intervention. A sub-sample of male and female participants from each study arm (~4 couples per arm) will participate in qualitative in-depth interviews to gain feedback about their participation in the intervention, including around processes of change (i.e., what pathways contribute to changes in alcohol and violence reduction) that might inform the content of future interventions. See Figure 2 for a diagram of study flow and data collection points.

### Study setting

The study will be conducted in Jaya Nagar slum in Bengaluru, India, where the research team has a history of engaging with a local community-based organization, Association for Promoting Social Action (APSA), through their self-help groups (SHGs). The chosen slum was selected in collaboration with this partner based on combined prior experience in the community and characteristics relevant to the proposed objectives of the pilot study that could ultimately contribute to the scalability of the intervention. These characteristics include low socio-economic status of residents, high levels of current alcohol use and IPV, social norms that demonstrate perceived acceptability of alcohol use and IPV, and inequitable gender norms.

### Randomization

Participants will be randomized at the couple-level prior to the start of the intervention by a statistician using a random number generator. Their randomization assignment will be revealed during an orientation session using a sealed envelope with their study ID.

### Study population

Our screening procedures will be designed to identify couples who would benefit from the study (i.e., those with problems related to alcohol and IPV), as well as to minimize the potential risk experienced by participants. The protocol achieves this by screening out men with severe alcohol dependence and experience of severe violence among women. The screening tool for men will utilize the AUDIT-C (23) to screen out non-drinkers and the Severity of Alcohol Dependence Questionnaire (SADQ) to screen out men with severe alcohol dependence syndrome. The SADQ was chosen based on its prior use by the World Health Organization (WHO) in multiple low-income countries, and its reliability and validity (24). During the screening process, men who score 31 or higher on SADQ and are deemed to be severely dependent will not be included in the study. Men who score as moderately dependent (16-30 on the scale) will be initially waitlisted until the team can determine the safety of enrolling these participants considering the possibility that these individuals may develop severe or complicated alcohol withdrawal syndrome requiring medical intervention. This subset of men will undergo a second medical screening by the study physician using the Clinical Institute Withdrawal Assessment for Alcohol Scale (CIWA-AR) (25) and those scoring above 8 on this scale will be excluded from the study.

While Standard WHO questions (26) on violence have been reliably used to measure violence in low and middle-income countries, no studies were identified that clearly documented procedures for screening out women experiencing severe violence whose risk may be increased by a couples-level violence prevention intervention. Following a risk averse model, the study team initially defined severe violence as the following types of violence occurring in the past two years: (a) being hit with a fist or something else that could hurt you on purpose, (b) being kicked, dragged, or beaten up on purpose, (c) being choked or burnt on purpose, and (d) being threatened or abused with a gun, knife, or other weapon. However, upon further discussion with stakeholders, our community-based partner, and pre-testing of the screening tool, we realized that these criteria would exclude most women experiencing violence and thus limit our ability to reach women in need of the intervention. As such, our screen-out criteria were adjusted to exclude women being kicked, dragged, or beaten up on purpose six or more times in the past 6 months, or those who were choked, burnt on purpose, threatened or abused with a gun, knife, or other weapon at any time in the past 6 months. Ever being hurt badly enough to require medical attention remained an exclusion criterion. All women who are screened out will receive access to referral resources for violence. In summary, the participant eligibility criteria include the following: (1) female partner between 18 and 40, (2) married, (3) speaks Kannada, (4) female partner reports male partner has a drinking problem, (5) female partner ever experienced psychological violence, and or physical, or sexual violence perpetrated by their male partner less than six times in the previous 6 months, and (6) both partners willing and able to enroll. Couples will be excluded if (1) the male partner is deemed to be severely alcohol dependent or at risk of severe alcohol withdrawal symptoms; (2) the female partner reported occurrences of severe physical or sexual violence characterized by attempting to choking or burning and threaten to use or actually used a knife or other sharp weapons against her or ever hurt badly enough that required medical attention; or (3) one or both members of the couple feared the counseling sessions may increase violence. Additional details regarding eligibility determination are described later.

### Safety procedures and ethical approval

Studies have shown that interventions targeting women experiencing IPV can result in an escalation of violence and the participants' safety being compromised (27). Based on our past experience in recruitment of women experiencing IPV, a multi-step recruitment process has been developed, with eligibility confirmation, provision of detailed information regarding the study and informed consent occurring at a designated community center, which provides a confidential and safe space. We initially designed to contact and consent female partners first in order to offer them the opportunity to weigh the risks and benefits of participation before providing consent and allowing the research team to contact their male partner. A similar approach was successfully implemented in a study recruiting mother-in-law's of young pregnant women experiencing abuse in Bengaluru (28).

In addition, a number of steps will be taken to protect participants against an escalation in physical and psychological violence and other risks. Alcohol and violence are stigmatized behaviors in communities despite their high prevalence (29, 30). Framing the study in terms of a family well-being intervention that aims to promote the well-being of families by reducing alcohol use, family conflict and violence, and increasing communication and support between the couple, including economic benefits as has been noted in earlier studies (31). In addition, we have developed a detailed case management and referral system that includes protocols for regular check-in calls with male and female participants over the course of the first 5 days of the intervention, as well as procedures for responding to physical and emotional health needs over the course of the study. The entire study team, from study case managers to community liaisons, will be trained in the identification of cues of emotional distress and signs of alcohol withdrawal, how to provide support to and make referrals for women and men who are experiencing IPV or alcohol withdrawal, and mandatory reporting procedures. Key staff from all referral agencies for women facing domestic violence will be sensitized to the study to facilitate improved trust and response for all referrals. During working hours, a medical doctor as part of the intervention team will be available to address any medical emergencies along with a trained counselor whose contact number will be made available to the men/women participants in case of any need. For support during after working hours, both men and women living in the same community where the study is planned will be identified as community liaisons to address any adverse event. The personnel will be trained to coordinate logistics between the hospital, participants and ambulance and also escort the men in case of alcohol related problems and women in case of any physical injuries to the hospital. Furthermore, we have identified shelter for women participants to stay in times of emergency related to an escalation in IPV.

Finally, the research team will be intensively trained on ethical issues in GBV research based on a team member's direct involvement in the development of global recommendations on GBV intervention research (32). Trainings are described in depth later in this manuscript.

This study has been approved by Institutional Ethics Committee, St. John's Medical College and Hospital, Bengaluru, India. All participants will be required to give their written informed consent prior to any study procedures.

## Study procedures

### Recruitment and enrollment

Couples will be recruited through outreach at women's SHG meetings, at community health camps, and through snowball sampling. The initial plan was to recruit female partners from SHGs run by our community-based partner prior to any male partner recruitment in order to avoid coercion of female partners into the study. However, despite reassurances that the study would not inform the male partner of the female partner's prior agreement, discussions with women revealed that they often desired male partners be contacted first. Therefore the recruitment plan was adjusted and research staff will use standardized scripts to describe the study and its purpose highlighting eligibility criteria, including male partner alcohol use. Interested individuals will be asked to indicate their interest in the study to the research staff, who will invite them to complete the informed consent process and an eligibility questionnaire. Eligible individuals will then be asked to provide contact information for their partners will be subsequently recruited. Once both members of the couple are deemed eligible, the couple will be enrolled in the study.

#### Intake process

Study staff will conduct all intake processes (i.e., informed consent and screening) at the community site. In instances where participants are not comfortable coming forward at the community site, staff will meet in an alternative private setting in the community.

### Study activities by arm

After being deemed eligible, couples will be asked to come to the study office for recruitment into one of the randomized intervention arms and an orientation session targeted for their specific study arm. During orientation, all couples will (1) learn more about the study specifics, (2) learn how to use the breathalyzer, and (3) complete a baseline survey. The baseline survey will cover basic socio-demographic information, gender norms, partner communication, relationship satisfaction, alcohol use, and IPV.

#### Comparison arm

Activities for the comparison arm will consist of prompts to men via cell phone messages to breathe into their breathalyzer once every other evening. Breathalyzer scores and a photo of the participant's face will be immediately recorded and stored in the Soberlink© system. The participation reward will be split into immediate earnings (which participants can come into the office to collect throughout the study) and savings (which will be deposited into their bank accounts as a lump sum at the end of the study). At the conclusion of the intervention period, men enrolled in the comparison arm will participate in a 60–90 min, community-requested alcohol education module focusing on couple's conflict management and economic goals.

#### Incentives arm

Couples enrolled in the incentives arm will participate in all of the activities described above for the comparison arm except that they will receive twice-daily prompts to breathe into their breathalyzer. Given our intention to combat IPV, which generally was posited to occur in the evenings when both partners are home, this pilot study incentivized sobriety in the evenings by scheduling daily breathalyzer tests in the evenings and early mornings. Couples will learn how the incentives are tied to breathalyzer scores and will be walked through a goal-setting exercise in which couples decide on goals they would like to save for such as children's education savings goals, individual savings goals, and business savings goals. Like comparison arm participants, daily and weekly activities for the incentives arm also consist of daily prompts to men to breathe into their breathalyzer via cell phone messages; however, these men will receive larger incentives for negative breathalyzer tests. Men will come in to the study office to receive their participation fees and a proportion of their breathalyzer test rewards, and will allocate another portion of the incentives to the goals they selected during the orientation session. A distinction was made between immediate rewards and long-term rewards for saving in our study design to allow men to feel that they were earning money for their behavior change that they could control, while also addressing a need to ensure the intervention contributed positively to family goals and shared financial decision-making with their female partners.

#### Incentives and counseling arm

Finally, those couples in the incentive and counseling arm will participate in all activities described for the incentives arm and also decide on a day of the week and time to come for BCT counseling. There will be a total of four sessions covering topics related to alcohol use, relationships, and communication. Each session will last for approximately 1 h. Sessions will be conducted by lay counselors who will be trained on BCT facilitation. Additionally, a clinical psychologist trained in CBT (ST) will supervise counselors and observe a subset of counseling sessions to monitor fidelity and measure quality of implementation.

While BCT is generally conducted over a significant duration of time rather than constrained to a discrete number of sessions, our pilot was designed to test the feasibility and resonance of key concepts and activities. The first BCT session will include an introduction to a “daily trust contract” during which the male partner verbally commits to reduce his drinking and his female partner acknowledges her support. The first session will also include a relaxation exercise to aid in overcoming the urge to drink. During the second session, the daily trust contract will be revisited and an activity to identify and avoid triggers to alcohol use on the way home will be introduced. Other strategies to avoid drinking will also be discussed, including the relaxation exercise, reviewing positive reasons for sobriety, distracting activities, and positive self-talk. The third session will include a review of prior alcohol avoidance strategies, as well as an activity aimed at encouraging caring behaviors between partners. In the final session, couples will review the caring behavior activity and will be introduced to the concept of active listening. All sessions will include role plays and end with assignments that couples will be asked to practice between sessions.

### Staff training and quality assurance

Prior to initiating this study, the local research team, composed of a study coordinator, interviewers and outreach workers, will attend a series of two training sessions. The first will consist of a 5-day research protocol training to familiarize the team with the study aims, protocol, and all research procedures. Research and intervention staff will be separated in their roles, however both will be familiarized with the overall study aims and procedures. Interviewer training will stress the importance of building rapport, active listening, asking questions with sensitivity and maintaining confidentiality of participants' responses. Study teams will receive information and training on reporting of social harms and how to assist or refer participants for emotional and social support, should any participant experience distress during an interview. Given the inclusion of alcohol use and IPV in these interviews and the selection of women who may likely have experienced violence, particular care will be taken to develop a close referral network of organizations providing alcohol de-addiction and IPV services through which participants can be referred. Additionally, all members of the research team will undergo research ethics training, which includes close attention to confidentiality and the protection of research participants. All trained staff, including members of our community partner, will be asked to take an oath indicating their commitment to protecting participant confidentiality.

The second training session for the study staff will be held just prior to the intervention component of the study and will consist of a 5-day training to again review the study aims, as well as train study staff in-depth on all intervention procedures, including the counseling.

### Data collection and management

Unique identification codes will be assigned to each data collection event and each study participant. These codes will be used to identify associated surveys, transcripts, breathalyzer data, as well as any individual-level information collected in data collection tracking forms. No names or other identifiable personal data will be collected in addition to the audio recordings or survey data and none will be associated with responses.

#### Breathalyzer data management

Data (time of test, photo, unique identification code, and BAC reading) will be directly transmitted to the SoberLink© system (web portal and secure server) which can be accessed only by selected team members. The SoberLink© system is US FDA approved. At the end of each week, a CSV file containing de-identified data including unique identification code, timestamp, and BAC score will be sent to the study team via email. Data will be reviewed for completeness and merged with survey data using the unique identification code.

#### Survey data management

Individual survey data collection will be done using tablets and sent electronically to a database for review on a regular basis. The use of electronic data collection on tablets will allow the data to be reviewed as it is collected. Each data collector will carry paper copies of the survey in case they confront problems with the tablet. If a survey must be completed on the paper form, the interviewer will reenter the data into his/her tablet immediately after returning from the field and resolving the issue with the tablet. To ensure confidentiality, the device will be password protected.

All local staff will be trained in maintaining participant confidentiality and rigorous data management based on standard operating procedures. All electronic files will be reviewed for completeness and accuracy.

#### Qualitative data management

All in-depth interview (IDI) guides will be translated to the local language (e.g., Kannada) and confirmed by a second qualified translator to ensure accuracy prior to conducting the interviews. IDIs will be recorded to ensure an audit of the content and process is possible. Permission for recording will be documented on the consent form. Only those who provide such permission will be included in the qualitative arm of the study.

IDI recordings will also be transcribed and translated into English (when needed) and typed into word processing files. These files will be uploaded into NVivo (or a similar qualitative software program) and thematically coded using a structured codebook, by trained analysts.

### Sample size

The sample size is anticipated to be 60 couples (20 per intervention arm). While this sample size is not powered to detect significance, it is similar to that used in our prior research of pilot interventions and is in line with recommendations that studies of this kind have a sample size of 24–50 (33–35). We anticipate attrition of approximately 20%, so our goal is to enroll 10–12 more couples to buffer these losses, yielding a final sample size of around 60 couples.

### Analysis

#### To evaluate the feasibility of implementing beautiful home

To evaluate implementation feasibility, we will calculate recruitment and retention rates and evaluate whether we had differential retention by study arm. We will stratify these assessments by arm to determine whether implementation feasibility differed, and will also measure contamination. If possible, we will also stratify assessments by level of alcohol use based on participants' breathalyzer scores. This will help us to determine whether participants with higher or lower alcohol use were more likely to complete the intervention.

#### To assess effects on safety and intermediate outcomes

Descriptive statistics will be calculated to describe participants' social and economic characteristics, relationship factors, gender norms and attitudes, and alcohol use risk behaviors. Descriptive statistics will also be calculated separately by intervention arm so that variations in distributions can be examined.

Most measures to be used in the analyses have been reported and validated in earlier studies. For some (e.g., gender norms), items will be grouped into scales conceptually and by examining item correlations through the use of factor analysis and other multidimensional scaling techniques.

We will examine whether there are systematic differences between individuals with missing data and those without. If missing data appear to be informative (e.g., missing systematically), we will employ appropriate ad hoc imputation methods to impute missing values.

#### Qualitative data analysis

In-depth interview data will be analyzed for dominant themes using grounded theory techniques. We will use textual data coding as a primary qualitative analytical approach to summarize, extract meaning, and condense the data, using NVivo qualitative software. Transcripts will be coded first through descriptive coding for key themes and topics, using a preliminary codebook. Additional codes will be identified through an iterative process of reading the textual data to identify emergent themes, and the codebook will be modified accordingly. Pattern codes, which achieve a greater level of abstraction, will be used to start linking themes and topics together to explore the relationship between various constructs described in the socio-ecological framework. (36) The findings and interpretations of the data will be critically discussed by the investigators until group consensus is reached on the dominant themes and meanings. Key findings will also be used to inform the refinement of the intervention and accompanying research materials for evaluation in a future randomized control trial.

#### Expected outcomes and results

This pilot study will provide information to determine the design of a future effectiveness trial. Specific outcomes include ability to recruit participants and retain couples across the three arms of the study. We will also assess the effects of intervention on household safety and male partner sobriety, and attitudes and behavior related to gender equality and shared household decision making. Results pertaining to outcomes will be available after the 3 month follow-up following the intervention. The study results will be presented at National and International Research meetings and published in peer reviewed scientific journals. Findings from this pilot study in favor of a follow-on trial will include the following: (1) the intervention is safe and feasible (i.e., no harmful events directly related to the intervention are detected); we are able to recruit participants in a timely manner; loss to follow-up is less than 20% overall and similar in all study arms; and no contamination is detected or if detected, appropriate design changes can be instituted; (2) data on outcomes support the impact of the intervention on alcohol use reduction and couples' communication skills; and trends in alcohol use and IPV incidence are in the direction that supports the positive impact of the intervention; and (3) qualitative evidence similarly supports the feasibility, safety and beneficial impact of the intervention. In conclusion, this study will generate important insights on a novel, urgently needed response to IPV in a high prevalence setting.

## Discussion

This protocol describes a pilot RCT of a novel intervention designed to reduce male alcohol use and IPV in an urban slum in India. The intervention, which was designed to build on evidence from the fields of behavioral economics and psychology, combines financial incentives and CBT to address and measure IPV in a way that, to our knowledge, has not previously been tested in low-income settings. While studies in low-income settings have tested both financial incentives and CBT, respectively for alcohol use and IPV, these interventions have yet to be tested in combination in these settings (9–11, 13, 20, 21).

The intention of the pilot is to identify lessons learned for the conduct of a full-scale RCT. With this in mind, procedures were designed to mimic those that would be used in the follow-on study. Critical safety procedures, including methods of describing the study's intention in the community, measures to screen individuals who may not benefit from the intervention, measures to minimize coercion of women during recruitment of couples, and a system of staff training and case management designed to capture and respond to all possible instances of harm were included. During study design, the adaptation of some of our designed approaches was crucial to respond to community needs and reach those who would benefit from intervention. As highlighted, we adjusted recruitment methods to allow for male recruitment prior to female recruitment. We also adjusted our threshold for defining severe violence, which was used to identify women who may be put at greater risk through participation. We took care to ensure that these adaptations maintained participant safety, particularly for women, while responding to the community context and needs. Given the relative novelty of working with couples to reduce IPV in low-income settings (14), this protocol offers guidance to researchers wishing to do so safely and will contribute to the development of global best practices.

## Conclusion

This paper reviews the study protocol for the *Beautiful Home* intervention, a three-armed randomized controlled pilot among couples in Bengaluru, India. The results of this pilot RCT will indicate whether a follow-on trial is warranted, as well as offer critical lessons for trial and intervention design. If preliminary results demonstrate that the intervention is safe and show positive improvements in male alcohol and IPV reduction, it could offer a potentially cost-efficient, scalable solution to IPV prevention in India.

## Ethics statement

This study was carried out in accordance with the recommendations of St. Johns Medical College & Hospital Institutional Ethics Committee. The protocol was approved by the St. Johns Medical College & Hospital Institutional Ethics Committee. All subjects gave written informed consent in accordance with the Declaration of Helsinki.

## Author contributions

SD, MH, KS, and VC conceived the study. MH, SD, KS, VC, and PA contributed to the development and refinement of the protocol. SS led the calculation of the sample size and quantitative analysis components of the protocol. All authors contributed to refinements in the scientific design of the intervention study. MH and QB drafted the protocol paper, and all the authors contributed to the draft and approved the final version of the paper.

### Conflict of interest statement

The authors declare that the research was conducted in the absence of any commercial or financial relationships that could be construed as a potential conflict of interest.
